# Paeoniflorin attenuates gestational diabetes via Akt/mTOR pathway in a rat model

**DOI:** 10.29219/fnr.v64.4362

**Published:** 2020-10-01

**Authors:** Yonghua Zhang, Yulin Liang, Huiqiao Liu, Ying Huang, Hongmei Li, Bo Chen

**Affiliations:** Department of Obstetrics and Gynecology, Heze Municipal Hospital of Shandong Province, Heze, Shandong, China

**Keywords:** GDM, mTOR, paeoniflorin, glucose, insulin

## Abstract

**Background:**

Gestational diabetes mellitus (GDM) is a type of diabetes associated with pregnancy and may impose risks on both mother and fetus. Akt paeoniflorin was shown to have anti-inflammatory and anti-hyperglycemia properties and has a potential ability to suppress mammalian target of rapamycin (mTOR) signaling. The current study aimed to study the effect of paeoniflorin on GDM maternal, fetal, and placental characteristics *in vivo*.

**Methods:**

Streptozotocin (STZ)-induced gestational diabetes rat model was used in our study. The expression levels of phosphorylation (p-) and total protein expression levels of protein kinase B (Akt), mTOR, serum/glucocorticoid regulated kinase 1 (SGK1), and eIF4E-binding protein 1 (4E-BP1) in the placenta were determined by Western blot assay. The blood glucose, insulin, and leptin levels were assessed using enzyme-linked immunosorbent assay (ELISA).

**Results:**

We found that placental Akt/mTOR signaling was substantially upregulated in GDM patients compared with healthy donors. Paeoniflorin administration alleviates the dysregulation of blood glucose, leptin, and insulin levels in both maternal and fetal GDM rats. Paeoniflorin treatment suppressed the overactivation of Akt/mTOR signaling in placental tissues. More importantly, administration of paeoniflorin was beneficial for normalization of fetal size and body weight in the GDM rats.

**Conclusion:**

Our study suggested that application of paeoniflorin may serve as a potential therapeutical strategy for patients with GDM.

## Popular scientific summary

- Paeoniflorin administration alleviates gestational diabetes mellitus associated syptoms, via suppressing certain signaling pathway involved in gestational diabetes mellitus.
- Paeoniflorin could be used for the treatment of gestational diabetes mellitus.

Gestational diabetes mellitus (GDM) is a common medical symptom that occurs in pregnant women (1). Approximately 1.7–12% of pregnant women may develop with GDM based on the different populations studied (2). During pregnancy, the placenta is the main organ to secrete various hormones, such as estrogen, progesterone, and prolactin, which exert insulin-antagonistic function. When the insulin secreted by the pancreas is not enough to control normal blood glucose levels, the blood glucose level rises, and the constant high levels of blood glucose contribute to the development of GDM (3). GDM is closely related to an increased frequency of fetal macrosomia, which may increase the incidence of development of cardiovascular disease, obesity, and diabetes for the descendant (4, 5).

Mammalian target of rapamycin (mTOR), an atypical serine/threonine kinase, is known to act as a master growth regulator that orchestrates diverse nutritional, chemical, and environmental signals such as growth factors, nutrients, energy exchange, and cytokines. mTOR presents in cytoplasm in two distinct complexes, which are mTOR complex 1 (mTORC1) and 2 (mTORC2) (6, 7). Insulin/PI3K/Akt signal transduction pathway acts as a master regulator of mTOR activity (8). Activation of mTOR promotes cell growth by phosphorylating its downstream targets, such as Unc-51 Like Autophagy Activating Kinase 1, 4E-BP1, p70S6 kinase (S6K), and SGK1 (9).

The placenta serves as a delivery system between the mother and the developing fetus (10). It provides fetus with nutrients and transfers waster products to the mother (11). The activity of Akt/mTOR signaling pathway in placenta has been proved to be associated with the nutritional, metabolic, and physiological state of the mother (12). mTOR can also affect the fetal growth through the regulation of placental development and function (13). Studies suggested that the placental mTOR is upregulated in pregnancies complicated by GDM, and mTOR overactivation is linked to fetal macrosomia (14, 15).

Paeoniflorin is a main biological active ingredient of a Chinese herb Paeonia lactiflora Pall. Many basic and preclinical studies suggested that paeoniflorin has potent anti-inflammatory, antitumor, and anti-hyperglycemia properties (16–19). Paeoniflorin exerts therapeutic effects on collagen-induced arthritis (*CIA*) rats via mediating Akt/mTOR signaling pathway and represses antibodies production from B cells (20). In addition, paeoniflorin imposes strong inhibition effect on mTOR-hypoxia inducible factor-1a pathway, leading to a reduction of alpha-smooth muscle actin (a-SMA) and collagen protein production and alleviation of liver fibrosis (21). However, the potential therapeutic effects of paeoniflorin on GDM women and their fetal development have never been explored.

In the current study, we focused on the investigation of the effect of paeoniflorin in streptozotocin (STZ)-induced diabetic rats fed with different doses of paeoniflorin. We aimed to answer the following questions: (1) Whether paeoniflorin oral administration ameliorates maternal body gain? (2) Whether paeoniflorin enhances insulin secretion and inhibits glucose and leptin upregulation? (3) What is the effect of paeoniflorin on fetal development? (4) Whether paeoniflorin suppresses the overactivation of placental Akt/mTOR signaling? The answers to these questions may assist us to have a better understanding of the effect of paeoniflorin on GDM women and their fetal development.

## Materials and methods

### Clinical samples

All participants were given a 75 g oral glucose tolerance test. The GDM is diagnosed if at least one value of plasma/blood glucose concentration is higher the thresholds of 92 mg/dL for fasting, 180 and 185 mg/dL for 1- and 2-h postglucose load glucose values, respectively. Placenta samples were obtained from 40 healthy women and 39 women with GDM after delivery. Samples were snap frozen and kept in liquid nitrogen. The Ethics Committee of Heze Municipal Hospital of Shandong Province approved this study, and all participants have given a written informed consent.

### Animal model and dietary formulas

Sixty-days-old female albino rats (Rattus norvegicus) were housed in standard polypropylene cages with free access to clean water and food, either normal diet or fatty-sucrosed diet. The Ethics Committee of Heze Municipal Hospital of Shandong Province approved the experimental protocol.

Rats were randomly divided into five groups (seven rats in each group) as indicated: (1) control: normal diet was given to rats for the entire study. (2) GDM: fatty-sucrosed diet was given to rats for the entire study. Five weeks later, rats were orally administered with the same amount of saline daily till the end of the experiment. (3, 4, and 5): GDM+paeoniflorin (5 mg/kg), GDM+paeoniflorin (15 mg/kg), GDM+paeoniflorin (30 mg/kg): rats fed with the fatty-sucrosed diet for 5 weeks. Thereafter, rats were orally administered with paeoniflorin (dissolved in normal saline; Sigma–Aldrich, St. Louis, MO) at a dose of 5, 15, and 30 mg/kg, respectively daily till the end of the experiment. All female rats were mated and got pregnant on week 5. The pregestational period indicates the whole-time period before mating, and the gestational period refers to the time period after mating. All GDM groups received intraperitoneal injection (i.p.) of STZ (25 mg/kg in citrate buffer; pH 4.5), and the control group was given a buffer i.p. injection.

### Enzyme-linked immunosorbent assay

The blood glucose levels were measured using a glucose assay kit (ab65333) from Abcam (Cambridge, MA, USA), and insulin secretion levels were determined using an insulin ELISA kit from RayBiotech (Norcross, GA, USA) according to the manufactory’s instruction.

### Western blot

The placental tissue samples were homogenized in ice cold radioimmunoprecipitation lysis buffer. Total proteins were separated using sodium dodecyl sulfate-polyacrylamide (SDS-PAGE) gel electrophoresis and were transferred to a polyvinylidene difluoride (PVDF) membrane (Millipore, Billerica, MA, USA). After blocking, the membranes were incubated with primary antibodies followed by incubated with secondary antibodies. The target protein signaling was detected using iBind western system (ThermoFisher Scientific, MA, USA). The antibodies against p-Akt, p-mTOR, p-4E-BP1, p-SGK1, t-Akt, t-mTOR, t-4E-BP1, t-SGK1, and b-actin were obtained from Cell Signaling Technology (Danvers, MA, USA).

### Statistical analysis

Data were expressed as mean ± SD. Two-way analysis of variance (ANOVA) with Bonferroni post hoc test was used to analysis the results of maternal blood glucose and insulin levels. Student’s *t*-test or one-way ANOVA with Bonferroni post hoc test was used to analyze the difference between the groups. The *P* value less than 0.05 was recognized as statistically significant.

## Results

### Placental Akt/mTOR signaling pathway was activated in GDM

Forty healthy women and thirty-nine women with GDM were enrolled in this study. As shown in Table 1, the pregestational body mass index (BMI), BMI at 34 weeks, and baby weight in GDM women were significantly higher than those in healthy women. The higher frequency of preterm delivery in GDM women compared with healthy women was observed. Women with diabetes family history exhibited a higher risk of GDM.

**Table 1:** Characteristics of study subjects

	Normal (*n* = 40)	GDM (*n* = 39)	*P*
Age at delivery (years)	29.1 ± 4.5	28.7 ± 5.1	0.713
Education level			
Lower than university	32 (80%)	32 (82.1%)	0.816
University or above	8 (20%)	7 (17.9%)	
Employed	27 (67.5%)	25 (64.1%)	0.750
Pregestational BMI	20.9 ± 2.2	23.7 ± 3.5	<0.001
Primiparous	21 (52.5%)	20 (51.3%)	0.914
BMI at 34 weeks	25.8 ± 2.4	29.2 ± 4.2	<0.001
Baby weight (g)	3,240 ± 125	3,620 ± 232	<0.01
Preterm delivery (<37 weeks)	2 (5%)	8 (20.5%)	0.038
Family history of diabetes			
Yes	5 (12.5%)	12 (30.8%)	0.048
No	35 (87.5%)	27 (69.2%)	

Values are means ± SD or number (percentage). BMI: body mass index.

**Fig. 1:**
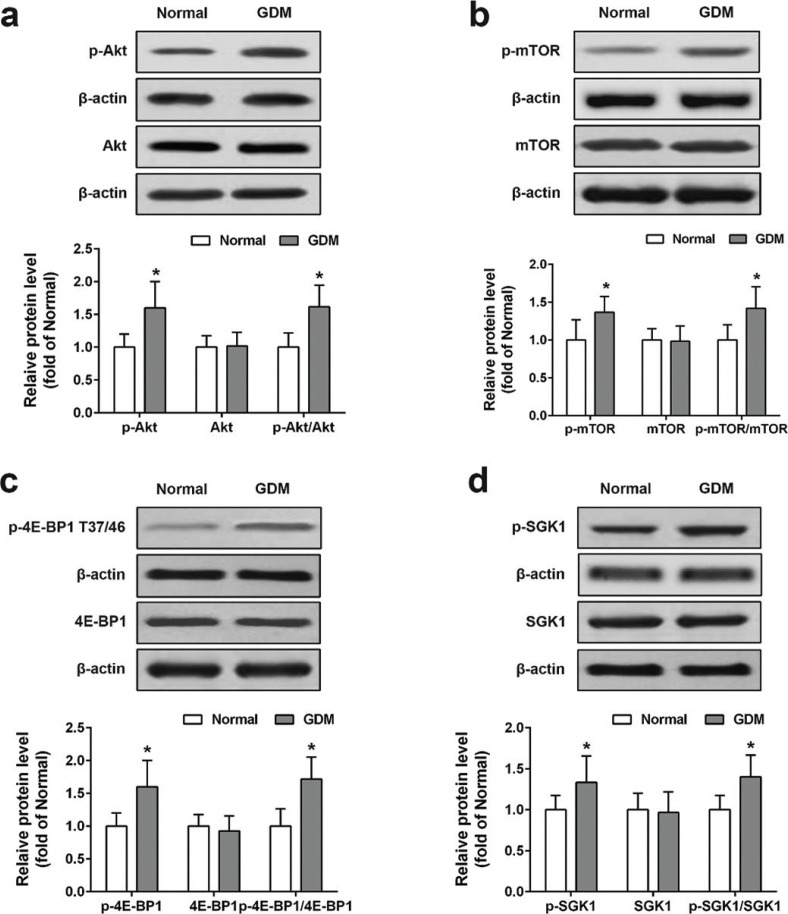
Expression of Akt/mTOR pathway-related genes in normal and GDM placental tissues. Proteins extracted from placental tissues were detected by Western blot, and relative protein levels of p-Akt, Akt (A), p-mTOR, mTOR (B), p-4E-BP1, 4E-BP1 (C), p-SGK1, and SGK1 (D) were analyzed. Data were means ± SD. *n* = 40 for normal group, *n* = 39 for GDM group. *P < 0.05 compared with normal group. Data were analyzed with one-way ANOVA followed by Bonferroni post hoc test.

To study the biological relevance of the Akt/mTOR signaling pathway in GDM, we compared the phosphorylation (p-) and total protein expression levels of Akt, mTOR, 4E-BP1, and SGK1 between normal and GDM placental tissues. We found a significant increase in the p-Akt, p-mTOR, p-4E-BP1, and p-SGK1 protein expression in GDM placental protein lysates compared with these in normal placental samples. However, the total Akt, mTOR, 4E-BP1, and SGK1 protein expression levels were not statistically different between normal and GDM groups (Fig. 1A–D). These results strongly suggested that overactivation of Akt/mTOR signaling pathway may play an important role in GDM development.

### Effect of maternal paeoniflorin diet on glucose, insulin, leptin, and body weight changes

Studies have implicated that paeoniflorin can elicit inhibition effect on mTOR signaling. We hypothesized that the inhibition of mTOR activation by administration of paeoniflorin might attenuate the GDM development. To verify this hypothesis, we used the STZ-induced diabetic rats fed with different diets (control, GDM, GDM+paeoniflorin [5 mg/kg], GDM+paeoniflorin [15 mg/kg], GDM+paeoniflorin [30 mg/kg]), as described in the ‘Materials and methods’ section.

**Fig. 2:**
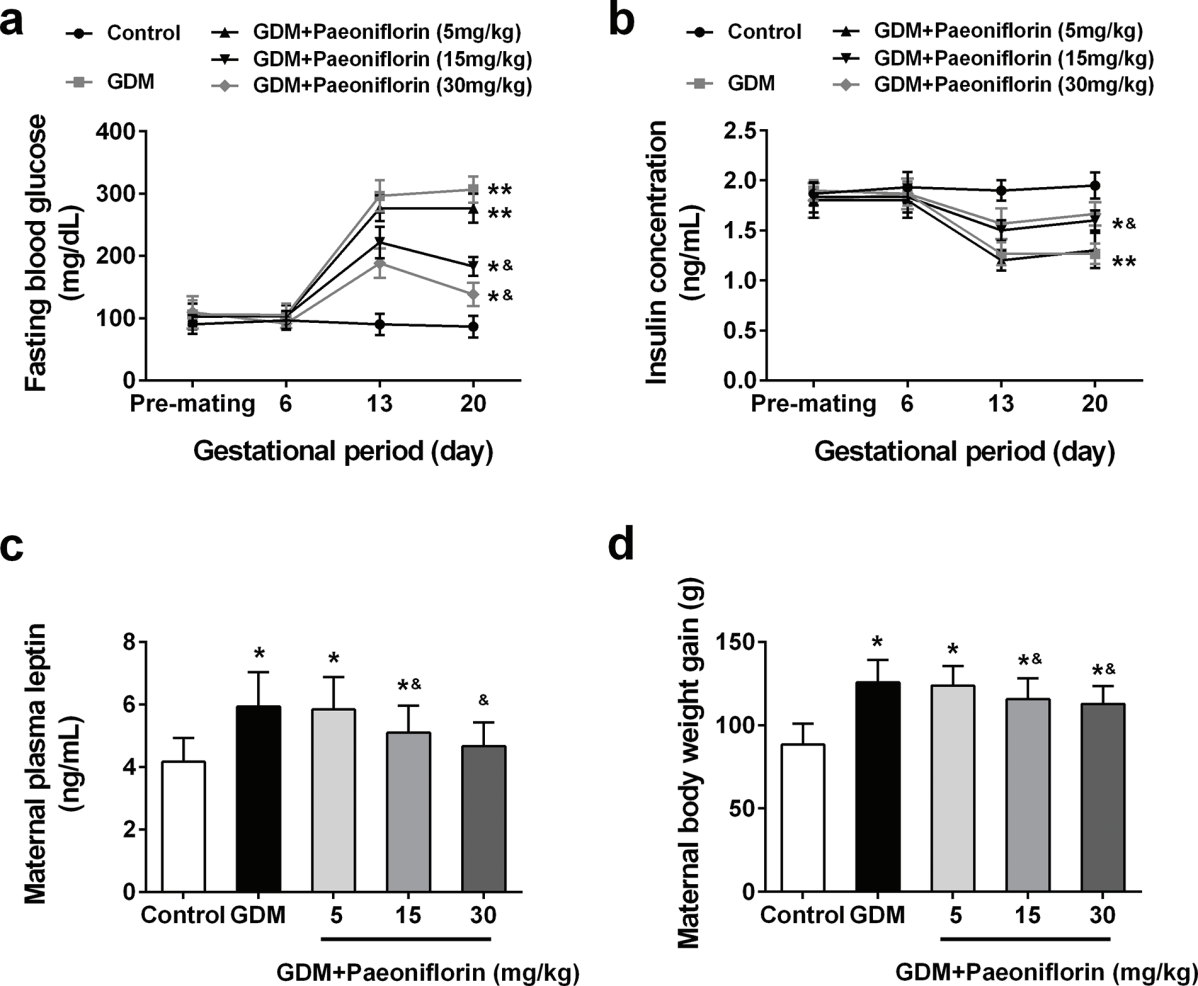
Effects of administration of paeoniflorin on maternal characteristics of GDM rats. Fasting blood glucose (A) and insulin levels (B) during the gestational period. Data were analyzed with two-way ANOVA followed by Bonferroni post hoc test. Maternal plasma leptin (C) and weight gain (D) at GD21. Data were analyzed with one-way ANOVA followed by Bonferroni post hoc test. Data were means ± SD. *n* = 7 for each group. *P < 0.05, **P < 0.01 compared with control group, ^&^*P* < 0.05 compared with GDM group.

Significant induction of blood glucose and plasma leptin levels was observed in all GDM groups compared with the control group. Paeoniflorin administration potentially alleviated the augmentation of blood glucose and plasma leptin in a dose-dependent manner in GDM groups (Fig. 2A and C). On the contrary, all GDM groups showed a marked reduction of insulin level compared with the control group (Fig. 2B). Interestingly, administration of paeoniflorin at 15 and 30 mg/kg, but not 5 mg/kg, elevated insulin levels in GDM groups with comparable ability. Figure 2D shows that all GDM rats fed with or without paeoniflorin exhibited a noticeable increase in their body weight gain as compared with control normal-fed rats. However, among GDM groups, oral administration of paeoniflorin evidently decreased their body weight gain in a dose-dependent manner compared with the dimethyl sulfoxide (DMSO) control-fed group.

### Effect of maternal paeoniflorin diet on litter size, fetal body weight, placental weight, fetal blood glucose, insulin, and leptin levels

**Fig. 3:**
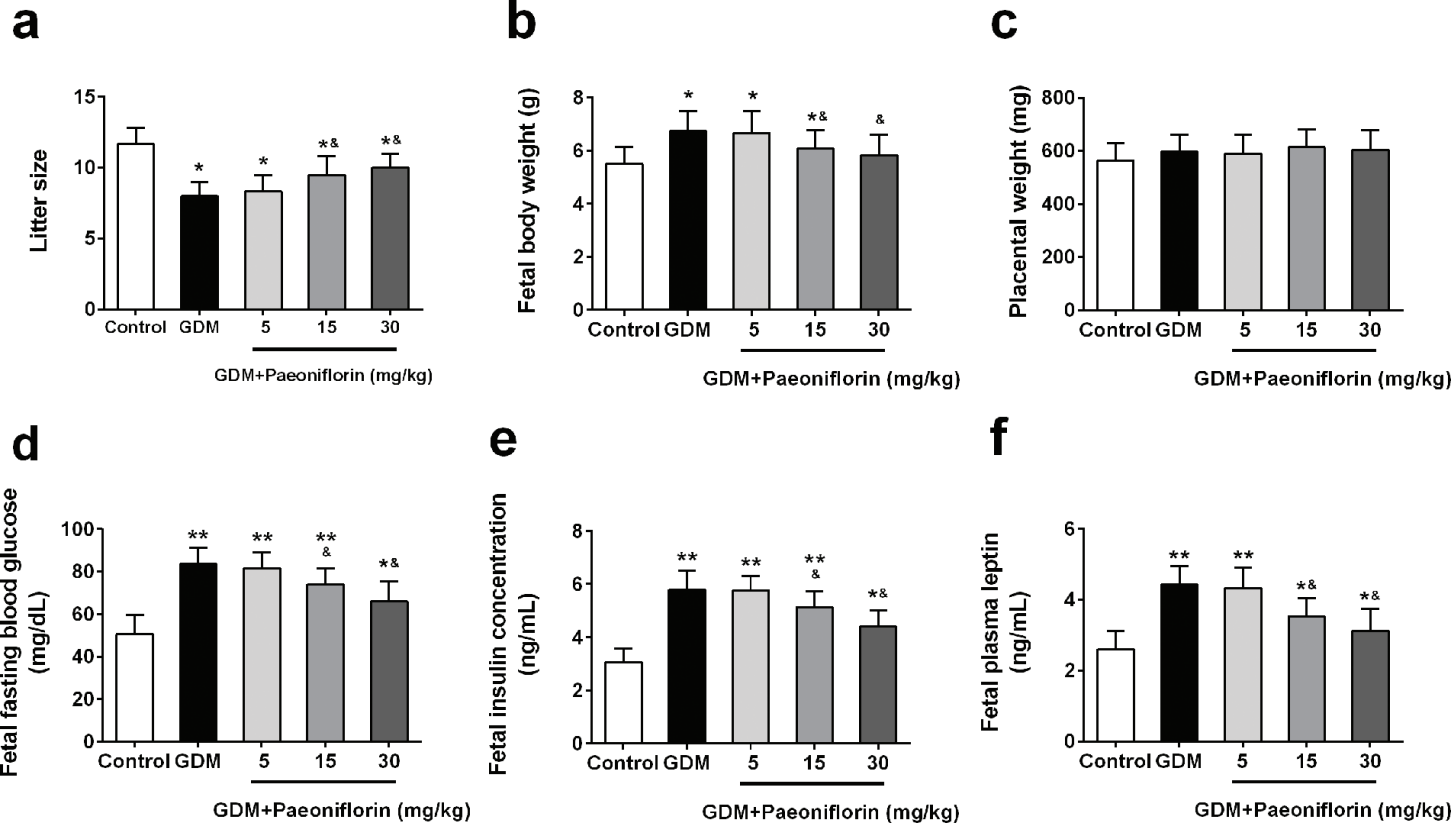
Effects of administration of paeoniflorin on fetal characteristics of GDM rats. Litter size (A), body weight (B), placental weight (C), fasting blood glucose (D), insulin levels (E), and fetal plasma leptin (F) were analyzed. Data were means ± SD. *n* = 7 for each group. *P < 0.05, **P < 0.01 compared with control group, ^&^*P* < 0.05 compared with GDM group. Data were analyzed with one-way ANOVA followed by Bonferroni post hoc test.

As illustrated in Fig. 3B, fetal body weight was significantly higher in all GDM groups than in the control group. Paeoniflorin administration at 15 and 30 mg/kg, but not 5 mg/kg, ameliorated the elevation of fetal body weight. In contrast with the fetal body weight pattern, the litter size was significantly smaller in all GDM groups compared with the control group and was elevated in paeoniflorin (15 and 30 mg/kg) administration groups compared with the DMSO group (Fig. 3A). There was no significant difference in placenta weight between groups (Fig. 3C).

As compared with the control group, fetal blood glucose, insulin, and leptin levels were dramatically upregulated in all GDM groups (Fig. 3D–F). Interestingly, paeoniflorin (15 mg/kg and 30 mg/kg) administration profound attenuated fetal blood glucose, insulin, and leptin levels when compared with the DMSO administration group.

### Effect of maternal paeoniflorin diet on Akt/mTOR signaling pathway

**Fig. 4:**
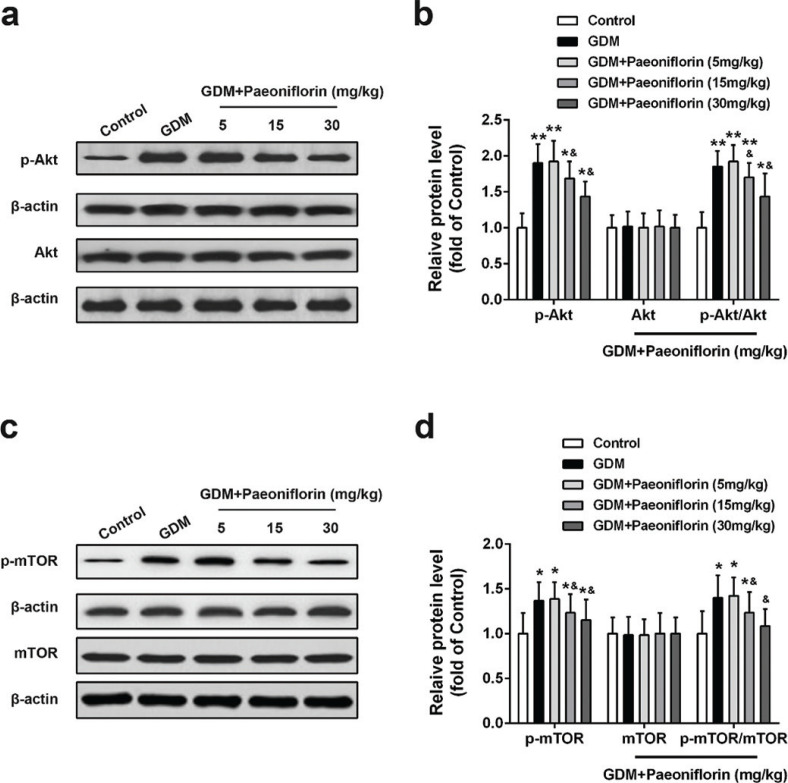
Effects of administration of paeoniflorin on Akt/mTOR pathway in normal and GDM placental tissues of rats. Protein levels of p-Akt, Akt (A, B), p-mTOR, mTOR (C, D) in placental tissues were detected by Western blot. Data were means ± SD. *n* = 7 for each group. *P < 0.05, **P < 0.01 compared with control group, ^&^*P* < 0.05 compared with GDM group. Data were analyzed with one-way ANOVA followed by Bonferroni post hoc test.

**Fig. 5:**
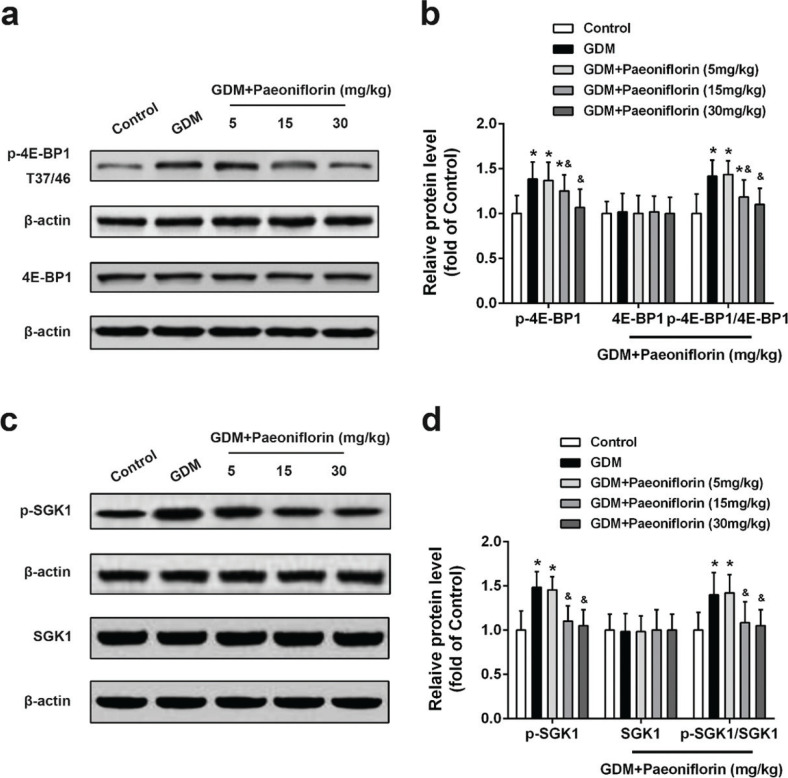
Effects of administration of paeoniflorin on proteins involved in mTOR pathway in normal and GDM placental tissues of rats. Protein levels of p-4E-BP1, 4E-BP1 (A, B), p-SGK1, and SGK1 (C, D) in placental tissues were detected by Western blot. Data were means ± SD. *n* = 7 for each group. *P < 0.05 compared with control group, ^&^*P* < 0.05 compared with GDM group. Data were analyzed with one-way ANOVA followed by Bonferroni post hoc test.

Similar to the changing pattern of fetal blood glucose, insulin, and leptin levels in Fig. 3, the expression levels of p-Akt, p-mTOR, p-4E-BP1, and p-SGK1 were robustly increased in the placental tissues of all GDM groups compared with the control group, as shown in Figs. 4A–D and 5A–D. Paeoniflorin (15 and 30 mg/kg) administration markedly attenuated the p-Akt, p-mTOR, p-4E-BP1, and p-SGK1 levels compared with the DMSO administration group. The total Akt, mTOR, 4E-BP1, and SGK1 protein expression levels were no difference between groups.

## Discussion

Previous studies have shown that the increased expression levels of p-Akt, p-mTOR, p-S6K, Adenosine 5’-monophosphate (AMP)-activated protein kinase (p-AMPKa), and p-4E-BP1 were observed in gestational diabetic placentas, highlighting the importance of Akt/mTOR signaling in GDM (22). Consistent with these findings, we confirmed that the expression levels of p-Akt, p-mTOR, p-4E-BP1, and p-SGK1 were profoundly upregulated in GDM placentas.

Because mTOR overactivation frequently occurs in the tissues of obese mice and humans. It has been suggested to play a vital role in the development of insulin resistance and type 2 diabetes (23, 24). The rapamycin, a well-known mTOR inhibitor, was widely tested in a number of animal models to improve metabolic parameters (25, 26). Unexpectedly, the rapamycin treatment shrank adipose tissue size and impaired β-cell function, causing deterioration of the metabolic profile (26, 27). One main reason is that rapamycin has profound deleterious effects on β-cell survival, proliferation, and function, and rapamycin may promote peripheral insulin resistance (28). Thus, a new method or strategy is urgently needed for the treatment of metabolic diseases, including GDM.

We explored the potential therapeutical application of paeoniflorin on GDM treatment using an STZ-induced diabetic rat model. Recent investigations of paeoniflorin suggested that paeoniflorin exhibited potent anti-inflammatory, antitumor, and immunoregulatory effects (16). Furthermore, paeoniflorin has been applied in preclinical studies for the treatment of rheumatoid arthritis (29), psoriasis (30), and systemic lupus erythematosus (31). Importantly, a recent study reported that paeoniflorin treatment led to reduced blood glucose levels, inhibited inflammatory factors section, and attenuated glomerular hypertrophy in diabetic rats (32). Similar to this finding, another study showed that paeoniflorin exerts a protective effect on vascular endothelial cells from hyperglycemia fluctuation-induced injury through reducing inflammatory reaction and oxidative stress (33). Our results demonstrated that oral administration of paeoniflorin at 15 or 30 mg/kg after mating and during the gestation significantly decreased the blood glucose and plasma leptin levels, and increased the insulin levels in rats with GDM.

The effect of paeoniflorin on fetal characteristics has never been investigated. Our study revealed that oral administration of paeoniflorin also exerts positive effects on maintaining normal fetal development. Paeoniflorin treatment on GDM rats markedly reduced the fetal blood glucose, insulin, and leptin levels to almost comparable levels with these in normal fetuses. These results suggested that paeoniflorin plays an important role in maintaining the normal fetal growth in GDM rats.

The underlying molecular mechanism of paeoniflorin-regulated GDM may relay on the fine-tuning of Akt/mTOR signaling pathway. Accumulating evidence has revealed that the Akt/mTOR signaling pathway plays a pivotal role in paeoniflorin-mediated numerous biological functions, such as arthritis, liver, and kidney diseases (34–36). For example, Li et al. showed that paeoniflorin mitigated the arthritis symptoms though the regulation of Akt/mTOR signaling to reduce antibodies production by B lymphocytes in *CIA* rats (34). Similarly, another study exhibited that paeoniflorin suppressed Akt activation to decrease Concanavalin A-induced IL-8 expression in human hepatic sinusoidal endothelial cells (35). Consistently, Hu et al. demonstrated that paeoniflorin blocked apoptosis of chondrocyte via the inhibition of IL-1β-induced Akt phosphorylation and cysteine-aspartic proteases (caspase)-3 activation (36). In line with others’ findings, we demonstrated a significant reduction of the expression levels of phosphorylation, but not total, Akt, mTOR, 4E-BP1, and SGK1 in the placenta of GDM rats.

Although the current data are exciting and promising, there is still a long way to go before these findings from rat of GDM model be translated into human clinical trial. For example, the optimal dose of paeoniflorin be used for rat and human is different and need to be addressed; the pharmacokinetic/pharmacodynamic model of paeoniflorin in GDM rat remains elusive; whether anti-inflammatory and antiapoptosis effects of paeoniflorin are involved in paeoniflorin-mediated alleviation of GDM symptoms, and what is the side effect of paeoniflorin to rat and human.

## Conclusion

Our findings indicated that overactivation of the Akt/mTOR signaling pathway plays a vital role in GDM development. Oral administration of paeoniflorin decreased the blood glucose levels and body weight gain in both maternal and fetal rats. We further revealed that paeoniflorin exerts beneficial effects on maintaining normal metabolic characteristics of the GDM placenta through fine-tuning of the Akt/mTOR signaling pathway. More animal experiments and clinical trials are urgently needed to evaluate the safety and efficacy of paeoniflorin for the treatment of GDM patients.
